# Supplementary material for: Thinking outside the CaaX-box: an unusual reversible prenylation on ALDH9A1
Source: RSC Chem Biol. 2023 Aug 30;4(11):913–25. doi: 10.1039/d3cb00089c (PMC10619140; doi:10.1039/d3cb00089c)
Supplement: CB-004-D3CB00089C-s001 [file CB-004-D3CB00089C-s001.pdf]

Electronic Supplementary Material (ESI) for RSC Chemical Biology.
This journal is © The Royal Society of Chemistry 2023

Supporting Information

Thinking outside the CaaX-box: an unusual reversible prenylation on ALDH9A1

Kiall F. Suazo,[a] Jakub Bělíček[b], Garrett L. Schey [c], Shelby A. Auger [a], Alexandru M. Petre,[a] Ling Li[d], Katarzyna M. Błażewska[e], David Kopečný[b] and Mark D. Distefano[a*]

[a]*Department of Chemistry, University of Minnesota, Minneapolis, MN 55455 USA*
[b]*Department of Experimental Biology, Faculty of Science, Palacký University, CZ-78371, Czech Republic*
[c]*Department of Medicinal Chemistry, University of Minnesota, Minneapolis, MN 55455 USA*
[d]*Department of Experimental and Clinical Pharmacology, University of Minnesota, Minneapolis, MN, 55455, USA*
[e]*Institute of Organic Chemistry, Faculty of Chemistry, Lodz University of Technology, Łódź, Poland*

Table of Contents

Figure S1. In-gel fluorescence analysis of GFP-ALDH9A1-expressing COS-7 lysates metabolically labeled with C15AlkCHO. 2
Figure S2. Extracted ion chromatograms corresponding the unmodified (A) and C15Alkthioester-modified (B) active site peptide of ALDH9A1. 2
Figure S3. Saturation curve for citral with ALDH9A1 3
Figure S4. MST binding curve for meldonium and TMABAL 3
Figure S5. Inhibition of ALDH9A1 with farnesal and citral 4
Figure S6. Structures of ALDH9A1 4
Figure S7. Possible structures involving Michael addition to C-3 of farnesal by Cys289 5
Figure S8. Electrostatic potential surface of ALDH9A1 5
Table S1. Fragment ions of the ALDH9A1 active site tryptic peptide obtained without and with thioester modification 6
Table S2. Kinetic constants of ALDH9A1 for citral 7
Sequences of primers used for mutation 7
References 7

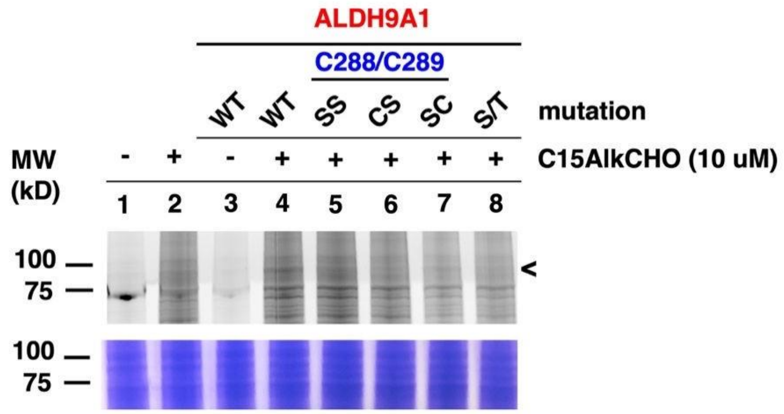

Figure S1. In-gel fluorescence analysis of GFP-ALDH9A1-expressing COS-7 lysates metabolically labeled with C15AlkCHO.

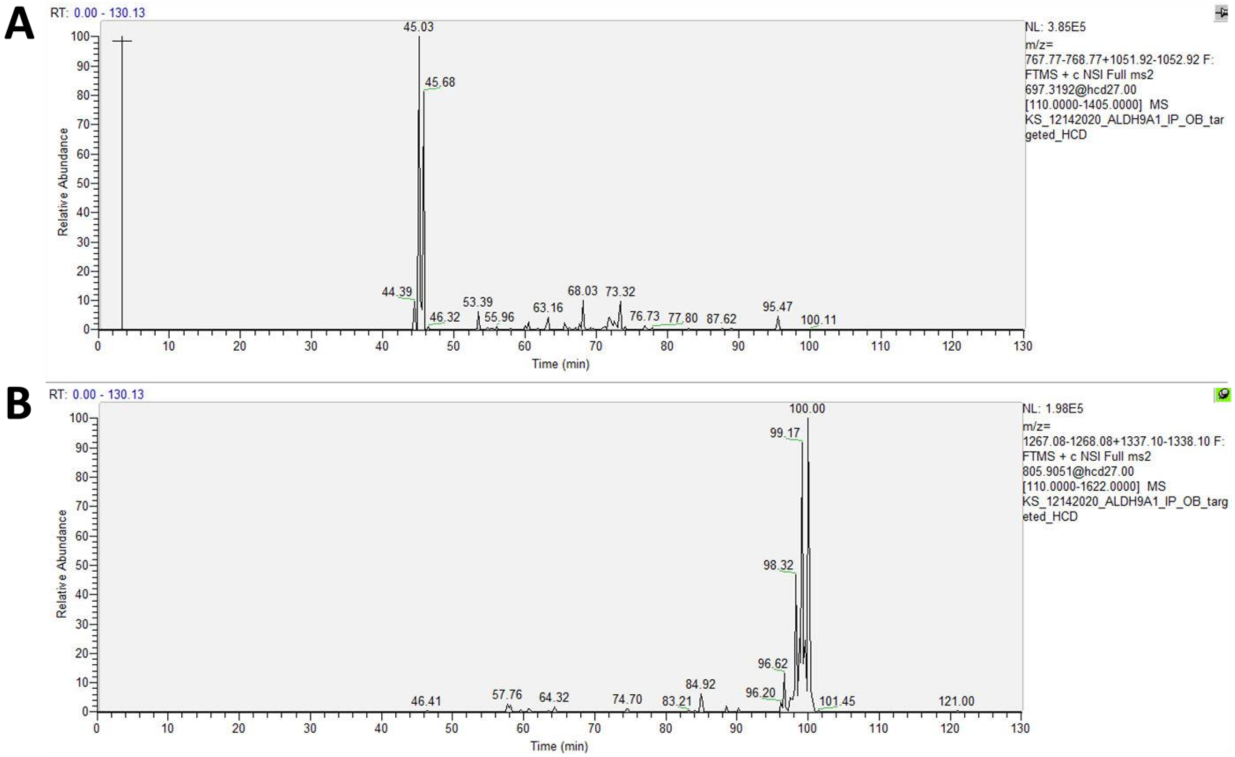

Figure S2. Extracted ion chromatograms corresponding to unmodified (A) and C15Alkthioester-modified (B) active site peptide of ALDH9A1.

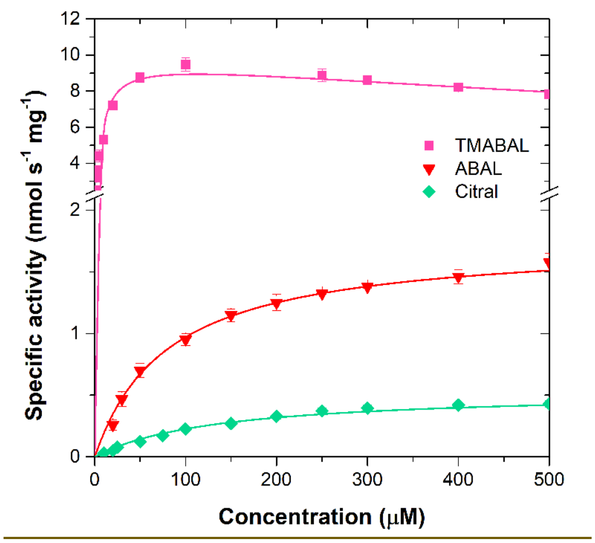

Figure S3. Saturation curve for citral with ALDH9A1. The data were measured using saturating 1.0 mM concentration of NAD^+ in 150 mM sodium pyrophosphate, pH 7.0 Curves for TMABAL and ABAL were previously published and are shown for comparison (Končitíková et al., 2019).

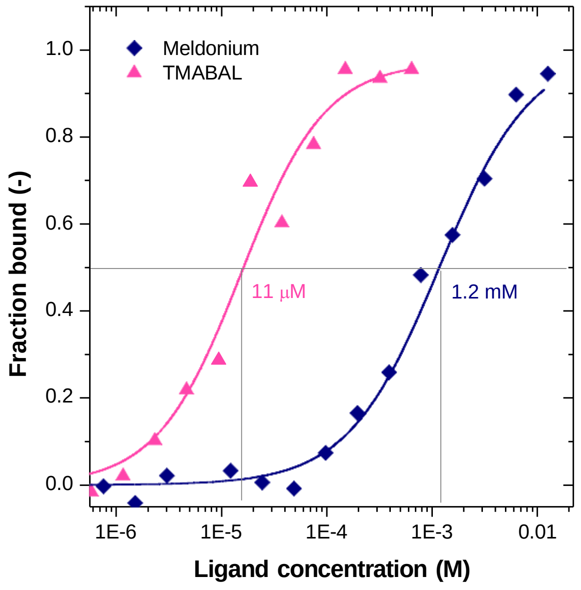

Figure S4. MST binding curve for meldonium and TMABAL. The ALDH9A1 was fluorescently labeled for the measurement. Binding curve of TMABAL is colored in pink, meldonium is in blue, K_D values are indicated.

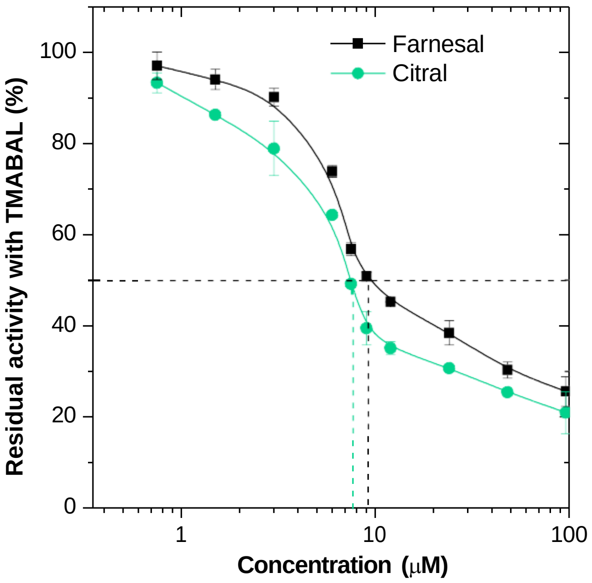

Figure S5. Inhibition of ALDH9A1 with farnesal and citral using TMABAL as a substrate. The data were measured with 6 μM TMABAL, 1.0 mM NAD^+ in 150 mM sodium pyrophosphate, pH 7.5 containing 1.0 mM NAD^+ at 30 °C.

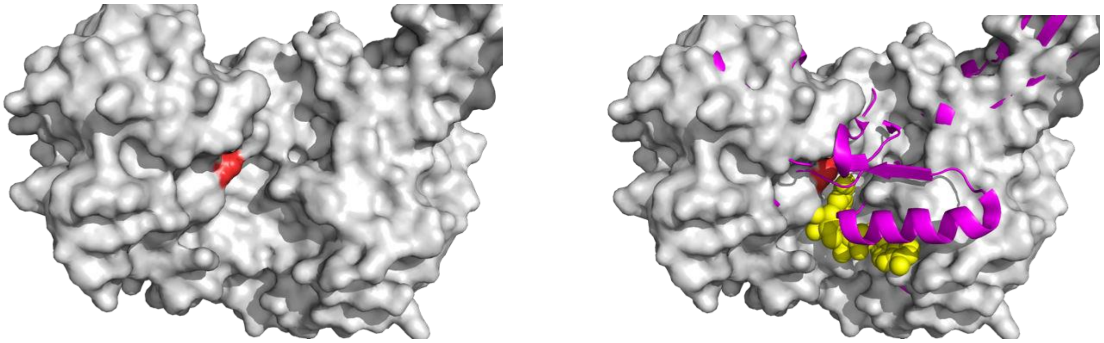

Figure S6. Structures of ALDH9A1. Left: Structure of enzyme in the absence of NAD+(pdb code 6QAP). Right: Structure of enzyme in the presence of NAD+ (pdb code 6VR6). Color scheme: enzyme in the absence of NAD+ (grey); Cys288 (red); Enzyme in the presence of NAD+(magenta); NAD+(yellow).

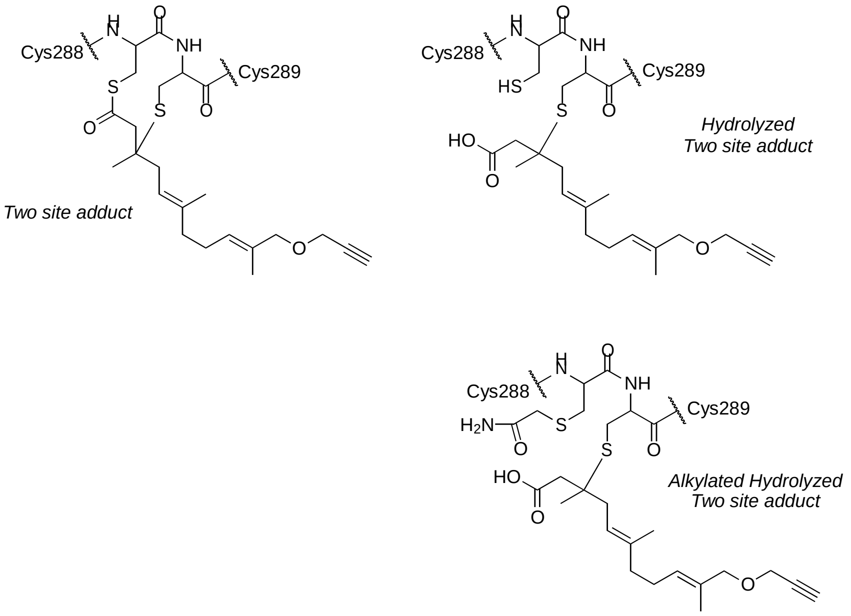

Figure S7. Possible structures involving Michael addition to C-3 of farnesal by Cys289. The "Two site adduct" would result from Michael addition prior to the proteomic work flow that includes iodoacetamide alkylation. If hydrolysis of the thioester occurred after the iodoacetamide alkylation, the "Hydrolyzed Two site adduct" would be present. If hydrolysis of the thioester occurred before the iodoacetamide alkylation step, the "Alkylated Hydrolyzed Two site adduct" would be present. None of these adducts were detected although it should be noted that the ionization of the observed thioester adduct was poor.

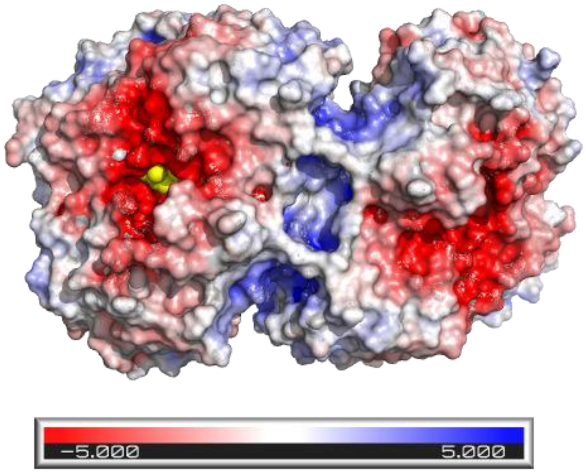

Figure S8. Electrostatic potential surface of ALDH9A1. The surface of ALDH9A1 (pdb code 6VR6) was calculated in Pymol and colored as noted in the legend. Farnesal (yellow) was docked into the active site and manually adjusted to bond with Cys288. ALDH9A1 is a dimer and this view looks down close to the C2 symmetry axis. Note that most of the farnesal group is sequestered by the enzyme and is hence not solvent accessible. The negatively charged surface in the vicinity of the bound farnesal is also noteworthy since it would be repulsive towards the membrane

Table S1. Fragment ions of the ALDH9A1 active site tryptic peptide obtained without and with thioester modification.

Ion Type	C288(Cam),C289(Cam)[a]			C288(C15Alkthioester),C289(Cam)[a]		
	m/z	intensity	m/z error	m/z	intensity	m/z error
b2+	215.1390	14.6832	-1.1526	215.1390	4.7479	-0.0835
b3+	343.1976	5.7959	0.3224	343.1976	1.7322	2.0123
b4+	400.2190	6.2064	-0.2760	400.2190	2.1554	2.0227
b5+	528.2776	10.2154	-0.4011	528.2776	3.6567	1.1133
b6+	627.3460	10.8375	-0.6066	627.3460	3.3977	2.1191
b7+	787.3767	1.9539	-0.1713	1002.5328	-	-
b8+	947.4073	1.0288	2.2280	1162.5635	-	-
b9+	1062.4342	3.9907	-1.8800	1277.5904	-	-
b11+	1220.5034	1.1269	0.3260	1435.6595	-	-
b11++	610.7553	0.5195	-16.2246	718.3334	-	-
y1+	175.1189	5.0454	-1.5034	175.1189	1.9810	-0.3613
y2+	276.1666	2.2817	0.1562	276.1666	1.6655	1.3511
y3+	333.1881	18.8114	0.0061	333.1881	15.4811	0.9365
y4+	448.2150	8.3148	0.0978	448.2150	10.3656	0.7002
y5+	608.2457	23.3810	-0.4940	608.2457	11.1590	0.6075
y6+	768.2763	91.2758	-0.7091	983.4325	11.1932	0.1629
y6++	384.6418	1.8886	-0.8076	492.2199	-	-
y7+	867.3447	60.3675	-0.6842	1082.5009	7.6610	-0.9131
y7++	434.1760	1.1442	-0.4260	541.7541	-	-
y8+	995.4033	22.1949	-0.6378	1210.5594	1.8784	0.0084
y8++	498.2053	1.8408	0.0187	605.7834	0.0000	0.0000
y9+	1052.4247	100.0000	-1.4500	1267.5809	10.4304	0.8907
y9++	526.7160	1.8801	-0.4770	634.2941	-	-
y10+	1180.4833	28.8661	-1.8953	1395.6395	1.7641	-0.9260
y10++	590.7453	66.1492	-0.2995	698.3234	0.7228	-3.6866
y11+	1281.5310	2.6356	-3.6058	1496.6871	-	-
y11++	641.2691	32.1587	0.1501	748.8472	-	-

[a]C288(Cam)C289(Cam) refers to the peptide where both cysteine residues have been alkylated with carboxamidomethyl groups at positions 288 and 289, respective whereas C288(C15Alkthioester)C289(Cam) refers to the peptide where Cys288 has a thioester and Cys289 has a carboxamidomethyl group.

Table S2. Kinetic constants of ALDH9A1 for citral. The data were measured with 1.0 mM NAD^+ in 150 mM sodium pyrophosphate, pH 7.5 at 30 °C. *The values for TMABAL and ABAL were previously published (Končitíková et al., 2019).

**Ligand**	K_m (µM)	V_{max} (nmol s^{-1} mg^{-1})	V_{max}/K_m (relative)
TMABAL*	6 ± 1	9.8 ± 0.4	100
ABAL*	67 ± 7	1.8 ± 0.1	1.6
Citral	136 ± 15	0.54 ± 0.03	0.2

Sequences of primers used for mutation

C288S/C289S:

Forward: 5’-AAT ACT CTT GTG CCA TTA GAG GAA ACC TGG CCT TGT GTG A-3’
Reverse: 5’-TCA CAC AAG GCC AGG TTT CCT CTA ATG GCA CAA GAG TAT T-3’

CS: C289S

Forward: 5’-ACA CAA GGC CAG GTT TGC TCT AAT GGC ACA AGA GTA TTG-3’
Reverse: 5’-AAT ACT CTT GTG CCA TTA GAG CAA ACC TGG CCT TGT GTG AG-3’

SC: C288S

Forward: 5’-CTC ACA CAA GGC CAG GTT AGC TGT AAT GGC ACA AG-3’
Reverse: 5’-CTT GTG CCA TTA CAG CTA ACC TGG CCT TGT GTG AG-3’

References

Končitíková, R.; Vigouroux, A.; Kopečná, M.; Šebela, M.; Moréra, S.; Kopečný, D. Kinetic and Structural Analysis of Human ALDH9A1. *Biosci. Rep.* **2019**, *39* (4), BSR20190558. https://doi.org/10.1042/BSR20190558.
